# Surgical outcome of upper extremity fractures in patients with Parkinson’s disease

**DOI:** 10.1038/s41598-020-78168-7

**Published:** 2020-12-03

**Authors:** Te-Feng Arthur Chou, Chun-Yao Chang, Jung-Pan Wang, Yi-Chao Huang, Wei-Ming Chen, Tung-Fu Huang

**Affiliations:** 1 Department of Orthopaedics and Traumatology, Taipei Veterans General Hospital, No. 201, Sec 2, Shi-Pai Road, Taipei City, 112 Taiwan, ROC; 2 Department of Orthopaedics, School of Medicine, National Yang-Ming University, No. 201, Sec. 2, Shipai Rd, Beitou District, Taipei City, 11217 Taiwan, ROC

**Keywords:** Outcomes research, Trauma, Neurology, Risk factors, Signs and symptoms

## Abstract

Patients with Idiopathic Parkinson’s Disease (PD) have an increased risk for fractures. Currently, many studies have reported inferior outcomes in PD patients after orthopedic procedures. However, there are very few studies assessing the outcome of upper extremity fractures (UEF) in PD patients. In this study, we reviewed 40 patients with PD that received surgical intervention for an UEF. We retrospectively reviewed patients with PD that received surgical fixation for an UEF at a tertiary trauma center. The primary objective was to determine the treatment failure rate after surgical fixation. The secondary outcomes include mode of failure, time to treatment failure, length of hospital stay, readmission rate, reoperation rate, and postoperative complications. A total of 40 patients with PD (42 fractures) underwent surgery. The most common fracture type was radius fracture (n = 19), followed by humerus fracture (n = 15), metacarpal/phalangeal fracture (n = 5), clavicle fracture (n = 2) and olecranon fracture (n = 1). The overall treatment failure rate was 40.5% (n = 17). The time to treatment failure was 1.24 ± 3.1 months and length of hospital stay was 6 ± 3.9 days, the readmission rate within 30 days was 14% (n = 6), and reoperation rate was 14% (n = 6). The complication rate was 16.6% (n = 7) and patients with humeral fractures appeared to have the longest hospital stays (6.6 days) and increased complication rates (13%, n = 2). Patients with PD have high treatment failure rates despite surgical fixation of an UEF. These patients often have a frail status with multiple comorbidities which may complicate their postoperative course.

*Level of evidence* level 4 case series.

## Introduction

Idiopathic Parkinson’s Disease (PD) is the most common neurodegenerative movement disorder. It is marked by gradual loss of dopaminergic neurons in the substantia nigra and pars compacta which leads to a reduction of dopamine levels^1^. It is characterized by resting tremor, bradykinesia, cognitive impairments, rigidity, dystonia, postural instability and unsteady gait^1^. Currently, PD affects 1–2 patients per 1000 of the population^2^. The prevalence is even higher in patients above 60 years as about 1% of this cohort is affected by PD^2^. Patients with PD are prone to falls and are at increased risk for fractures, with fractures involving the hip, spine and forearm being the most common^3,4^. Due to the frail status of PD patients, falls cause significant morbidity which leads to impairment of daily function^4^. Currently, there have been several studies that assessed the outcome of orthopaedic procedures for PD patients^4–6^. In particular, studies that evaluated surgical fixation for fractures of the hip and degenerative spinal disorders have all revealed higher rate of revision surgeries and postoperative complications^6–8^. Oichi et al. performed a matched-pair cohort study of 1423 PD patients and noted a significantly higher crude in-hospital mortality (0.8% vs. 0.3% in controls) and crude proportion of major complications (9.8% vs 5.1% in controls) after spinal surgery in patients with PD^8^. Meanwhile, Galbusera et al. noted a higher rate of postoperative complications and revision surgeries in PD patients after spinal surgery^7^. In addition, surgical fixation for hip fractures in PD also showed increased rates of postoperative complications and inferior functional recovery in comparison to non-PD patients^6,9^. Therefore, many experts have concluded that PD may be a risk factor for inferior outcomes (e.g. delayed functional recovery, longer hospital stays, and increased revision surgeries) in comparison with non-PD patients after orthopaedic procedures^6,7,10,11^. In current literature, there are very few studies evaluating the outcome for upper extremity fractures (UEF) that occurred in PD^12–15^. Our team recently reported the outcome of distal radial fractures after surgical fixation for PD^12^. In agreement with fractures that occurred in other bones, PD patients had a significantly higher treatment failure rate despite appropriate surgical management of distal radial fractures. In this study, we reviewed 40 patients with PD that had an UEF that was managed surgically at our institution. Our aim is to determine the surgical outcome of PD patients and to review the current literature on UEF in PD. Specifically, we want to identify the treatment failure rate (e.g. malunion, nonunion, persistent pain over fracture site) of PD patients that underwent surgical fixation for UEF. In addition, we also would like to identify the incidence of potential risk factors for failure.

### Methods

 This was a retrospective review of PD patients that underwent surgical fixation for an UEF. All of the patients were managed and treated surgically at a tertiary trauma center in Taipei, Taiwan. The study was approved by the institutional review board of Taipei Veterans General Hospital and informed consents were obtained from each patient. Human research guidelines were in accordance with guidelines provided by the World Medical Association declaration of Helsinki. We defined UEF as a fracture of the clavicle, humerus, ulna, radius and hand. The primary outcome was to assess the treatment failure rate of PD patients that underwent surgical fixation for UEF. In addition, we also recorded the type of UEF, the treatment received, the mode of failure, time to treatment failure, length of hospital stay, readmission rate within 30 days, reoperation rate, and postoperative complications (e.g. pneumonia, urinary tract infection, delayed wound healing).

### Study population

We retrospectively reviewed the medical records of all the patients with PD complicated with UEF that received surgical intervention at our institution. A diagnosis of PD was made based on clinical history, review of medical records (ICD-9 CM code 332.0) and identifying PD-related drugs in the prescription records (levodopa-carbidopa, dopamine agonists, monoamine oxidase B inhibitors, catechol-O-methyltransferase inhibitors). In terms of UEF, we included all fractures involving the clavicle, humerus, forearm and hand fractures. The fractures were classified according to the Arbeitsgemeinschaft für Osteosynthesefragen (AO) surgical reference guide for orthopedic trauma^16^. The following patients were excluded from our study: patients under the age of 18, patients that were managed conservatively, and open or complex pathological fractures. We excluded patients treated with conservative treatment mainly because of two reasons. First, given the high prevalence of osteoporosis in PD, patients that were treated with conservative treatment frequently had malreduction which could have significantly increased the failure rates in these patients. In addition, most patients in this subgroup did not receive surgery because of debilitating comorbidities and were unfit for surgery. Therefore, this may also negatively impact the results. As for open and/or complex pathological fractures, these fractures have several factors (e.g. infection, staged-surgery and inadequate fixation due to comminution of the fracture) that may also negatively impact the outcome. Therefore, these patients were excluded, and only relatively simple fractures were included in this review.

### Surgical intervention and patient assessment

All of the included patients underwent surgical reduction and fixation for an UEF. All surgeries were performed by fellowship trained orthopaedic surgeons. The fixation of fractures were achieved either with external fixation, kirschner wire, plating, intramedullary nailing, hemiarthroplasty or a combination of the above. The choice of fixation method was determined based on the fracture pattern and the surgeon’s preference. For each patient, we recorded the American Society of Anesthesiologist physical status grade, history of coronary artery disease, diabetes mellitus, chronic kidney disease, osteoporosis, and smoking status.

The standard postoperative follow-up protocol includes patient visits at 2 weeks, one month, three months and 12 months after the surgery. We removed the stitches around 2–3 weeks after the surgery depending on the wound condition. Immediately after the surgery, the patient was immobilized either with a protective arm sling or a supportive splint after the surgery for one month for precaution measures. At postoperative 1 month, the sling or splint was removed and rehabilitation was initiated with range of motion exercises specifically for the injured site. At three months of follow-up, if the patient continued to have persistent stiffness or subjective limitation with range of motion, we referred the patient to a physical therapist to initiate therapy. If treatment failure was noted prior to the final follow-up, the follow-up duration was recorded as the time of treatment failure. Standard posteroanterior and lateral radiographs were obtained immediately after the surgery and at each visit. For radiographic assessment, we assessed for union of fracture, screw position, and bony alignment. Treatment failure had variable definitions which was determined based on the type of fracture. In general, the presence of a fracture line beyond 6 months after the surgery, loss of reduction, or persistent tenderness upon palpation of the fracture site without secondary causes was considered treatment failure. All measurements and assessments were completed by senior orthopaedic surgeons. All quantitative data were expressed as mean ± SD and categorical data were recorded as percentages.

### Results

After exclusion, a total of 40 patients (42 surgeries) were enrolled in this study. The mean age was 74 ± 8.5 years-old and the percentage of female patients was 59.5%. The mean disease duration was 16.3 ± 2.1 months and the mean follow-up duration was 5.3 ± 3.1 months. The baseline characteristics are shown in Table 1. Of note, 65% (n = 26) of the patients were prescribed with levodopa at the time of injury. There were five fracture types that occurred in our patients, including clavicle, humerus, olecranon, radius and metacarpal/phalanx fractures.

**Table 1:** Baseline characteristics.

	Patients with PD (n = 42)
Age	74 ± 8.5 years old
Female patients (%)	25 (59.5%)
**ASA class**	
Class I/II	10 (23.8%)
Class III/IV	31 (73.8%)
Not recorded	1 ( 2%)
**PD disease duration (months)**	16.3 ± 2.1
Levodopa use	26 (65%)
Follow-up duration (months)	5.3 ± 3.1
Length of stay (days)	5.95 ± 3.94
Smoking history	2
**Comorbidities**	
Coronary artery disease	9
Diabetes mellitus	6
Chronic kidney disease	3
Osteoporosis	17

*ASA* American Society of Anesthesiologists, *PD* Parkinson’s disease.

**Figure 1:**
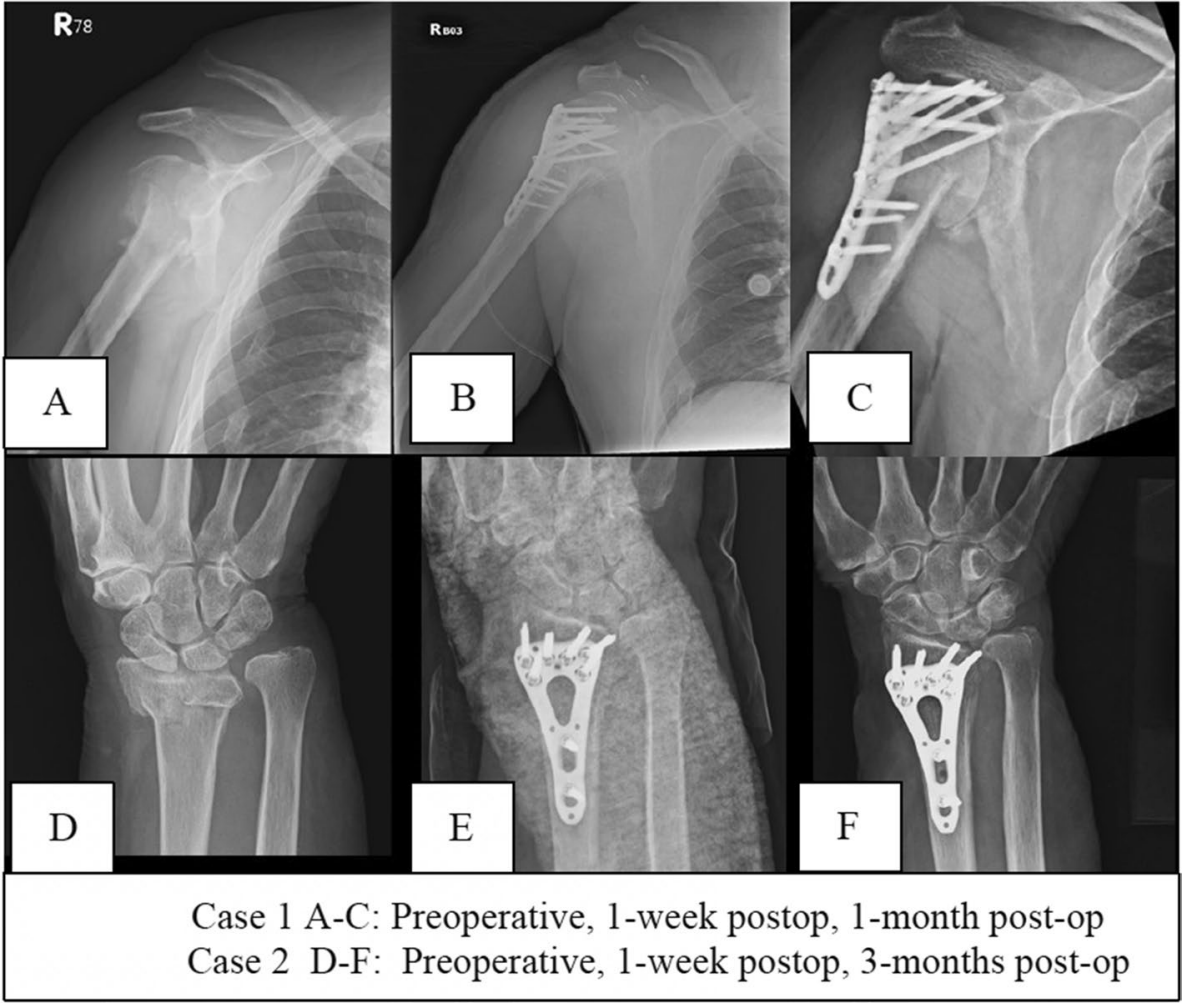
(**A**–**C**) This was a 75-year old male that presented with right proximal humerus fracture treated with AO-philos locking plate. Postop x-rays: revealed appropriate fixation and alignment. However, loss of reduction was noted 1 month after the surgery and eventually screw penetration was noted which required removal of implant at three months after the surgery. (**D**–**F**) A 67-year old female that received plating for a left distal radius fracture. Progressive loss of radial height and eventually screw penetration was noted, violating the wrist joint which required removal of implant.

The surgical method and outcome for each fracture type is shown in Table 2 and Table 3, respectively. For most of the patients, plating was used for surgical fixation (n = 25, 60%), followed by k-wire fixation (n = 11, 26%), external fixation (n = 4, 10%), while one patient had joint replacement and another patient received intramedullary nailing (n = 1, 2%). A total of 17 patients had treatment failure (40.5%) and the most common mode of failure was loss of reduction (n = 8, 47.1%), followed by malunion and nonunion (n = 6, 35.3%). Two patients that had typical failure patterns are presented in Fig. 1. The average time to treatment failure was 1.24 ± 3.1 months. The mean length of hospital stay was 6 ± 3.9 months. The rate of readmission within 30 days was 14% (n = 6), and 14% (n = 6) of the patients underwent revision surgeries within 3 months after the surgery. In terms of postoperative complications, postoperative delirium (n = 4) was the most commonly seen complications, followed by pneumonia (n = 2) after the surgery which required re-hospitalization for medical treatment, while one patient had delayed wound healing which was complicated with a superficial bacterial infection. Finally, 3 patients required postoperative rehabilitation due to suboptimal recovery of range of motion of the injured extremity.

**Table 2:** Surgical methods.

	Clavicle	Humerus	Olecranon	Radius	Metacarpal/phalanx	Overall
Number of surgeries	2	15	1	19	5	42
Female patients (%)	2 (100%)	8 (53.3%)	1 (100%)	13 (68.4%)	3 (60%)	25 (59.5%)
**Surgical intervention**						
Kirschner wires	0	0	1	5	5	11 (26%)
External Fixation	0	0	0	4	0	4 (10%)
Plate	2	13	0	10	0	25 (60%)
Nail	0	1	0	0	0	1 (2%)
Joint replacement	0	1	0	0	0	1 (2%)

**Table 3:** Perioperative outcome.

	Clavicle	Humerus	Olecranon	Radius	Metacarpal/phalanx	Overall
**Outcome**						
Union	1 (50%)	9 (60%)	1 (100%)	13 (56.5%)	1 (20%)	25 (59.5%)
Treatment failure	1 (50%)	6 (40%)	0	6 (40%)	4 (80%)	17 (40.5%)
**Mode of failure (n** **=** **17)**	1	6	0	6	4	
Nonunion/malunion	1	1	0	3	1	6 (35.3%)
Loss of reduction	0	4	0	2	2	8 (47.1%)
Persistent pain	0	1	0	0	0	1 (5.9%)
Infection	0	0	0	1	0	1 (5.9%)
Others	0	0	0	0	1	1 (5.9%)
Time to failure (months)	0.5	1.2	0	1.1	1.7	1.24 ± 3.1
Length of stay (days)	4.5	6.6	6	5.53	6.2	6 ± 3.9
Readmission (< 30 days)	1	2	0	3	0	6 (14%)
Reoperation	1	2	0	1	2	6 (14%)
**Complications**	0	2	0	1	0	7 (16.6%)
Pneumonia	0	2	0	0	0	2
Urinary tract infection	0	0	0	0	0	0
Wound	0	0	0	1	0	1
Mortality (30 day)	0	0	0	0	0	0
Thromboembolism	0	0	0	0	0	0
Postoperative delirium	0	2	1	1	0	4

### Discussion

In this study, we reviewed 40 patients (42 fractures) that presented with an UEF and were surgically managed at a tertiary trauma center. The main finding was the high failure rate for PD patients after surgical treatment of UEF. To our knowledge, this is one of the few studies that assessed the surgical outcome of UEF in PD. Previous studies have focused on the incidence and outcome of spine instrumentation surgery and surgical fixation of hip fractures, with very few studies assessing UEF. In our study, we noted a treatment failure rate of 40.5% in PD, which is very high when compared with the results presented in current literature. Overall, the treatment failure (e.g. nonunion, malunion etc.) rate of UEF in the general population is reported to be around 0.2–24%^17–20^. Factors that may predispose to failure include site of fracture, type of surgical treatment and fixation method, fracture displacement, treatment delay, smoking and infection^21^. With regards to the mode of failure, loss of reduction was the most common pattern. Of the 8 patients that had loss of reduction, humeral fractures (n = 4) were the most common fracture type. In current literature, osteoporosis is a well-documented risk factor for humeral fractures^9^. PD patients frequently have severe osteoporosis leading to fragile and insufficient bone quality^22^. In a review performed by Raglione et al., the authors noted a major reduction of bone mass density in PD patients when compared with age-matched cohort^22^. Therefore, severe osteoporosis in this population may have led to increased rates of failure. For hip fractures, some surgeons have favored the use of joint replacement surgeries for PD patients^4,9^. In UEF, Kryzak et al. previously reviewed the outcome of shoulder hemiarthroplasty in PD patients after humeral fractures^13^. Their results only showed marginal benefits, with three out of the seven patients having persistent pain and limited functional recovery after the surgery^13^. In a large series conducted by Burrus et al., the authors noted increased rates of infection, dislocation, revision surgeries, fracture, component loosening, and systemic complications after shoulder arthroplasty^15^. In our series, only one patient received hemiarthroplasty of the shoulder. This patient was complicated with postoperative pneumonia which required extended hospital stay for antibiotics treatment. She was able to retain her prosthesis, and had adequate pain relief. However, she only had minimal functional recovery which is to be expected with a shoulder hemiarthroplasty^13^.

The second most common mode of failure was nonunion or malunion. Primary, DRFs have a very low nonunion or malunion rate (0.2–10%), but this study revealed very high failure rates for PD patients (15.8%)^12,18^. In a previous study conducted by our group, the treatment failure rate after DRF in PD can be as high as 39%, with loss of reduction and malunion of fracture being the two most frequent mechanisms^12^. When compared with non-PD patients, k-wires and external fixation appeared to have inferior results for PD, while plating of the radius had similar success rates. Since PD patients have rigidity, tremor and unsteady gait, absolute stability of the fracture should be achieved in order to facilitate healing. Therefore, fixation methods that achieve absolute stability such as plating should be considered when treating these patients^12^.

Interestingly, fractures of the hand, including metacarpal (MCP) and phalangeal fractures had poor results after surgical fixation in this population. Currently, most of the fractures of the hand can be managed with k-wire fixation with satisfactory results^23^. In this study, four of the five fractures of the hand (4 MCP and 1 phalangeal fracture) did not heal as anticipated. There were two patients that had loss of reduction, one patient with nonunion, while one patient had k-wire penetration into the proximal interphalangeal joint, which required removal of the k-wire 2 weeks after the surgery. In a prospective study performed by Strub et al., conservative treatment for fracture of the fourth and fifth MCPs appear to have similar functional results in selective patients when compared with patients that were treated surgically^24^. Given the relatively lower physical demands and suboptimal results after k-wire fixation in PD patients, conservative treatment either with cast or protective splint should be considered for these patients.

One concern for PD patients is the limited improvement in functional performance after surgery. Previous studies have concluded that PD patients have inferior functional recovery after spinal instrumentation surgery and hip surgical fixation, while patients that underwent shoulder hemiarthroplasty only had minimal functional improvements^4,7,13^. This can be attributed to the increased muscle tone, severe tremor, and poor gait stability^4^. The increased muscle tone and frequent tremors may act as a destabilizing forces to the fracture, causing constant motion at the fracture sites impeding proper fracture healing. In our study, patient reported outcomes (e.g. Disabilities of the Arm, Shoulder and Hand questionnaire; the Patient-Rated Wrist Evaluation, Mayo Wrist Score) were not consistently recorded after the surgery^25^. Therefore, a conclusion as to the functional recovery of UEF remains to be determined. On the other hand, gait instability and poor postural balance can further cause a second fall leading to subsequent injuries. Of the 40 patients, two patients had a second fall during their recovery course (< 3 months postoperatively), leading to fractures of the hip. Therefore, strong social support and strict fall precautions should be provided to prevent such injuries.

Our study has several limitations that need to be addressed. This was a retrospective review of 40 patients with PD that was treated surgically for UEF. Due to the nature of the study design and relatively small sample size in each fracture type, it was difficult to draw significant conclusions. Additionally, we did not have a comparison group to assess for statistical significance. To overcome these limitations, a comprehensive literature review was completed for the available evidence on UEF in the general population and was used to compare with our results. Lastly, we did not include patients that received conservative treatment in this study. Future studies should include a comparison group as well as patients that underwent conservative treatment. Nonetheless, the difference in failure rates were quite striking, and should alarm the physician that these patients may have a high tendency for failure of fixation despite appropriate treatment.

### Conclusion

Patients with PD have a high treatment failure rate (40.5%) after surgical fixation of an UEF. The most common mode of failure is loss of reduction. Since PD patients often have multiple comorbidities, extended hospital stays and complex postoperative course should be expected.

## References

[1] Maiti P, Manna J, Dunbar GL (2017). Current understanding of the molecular mechanisms in Parkinson's disease: targets for potential treatments. Transl. Neurodegener..

[2] Tysnes OB, Storstein A (2017). Epidemiology of Parkinson's disease. J. Neural Transm. (Vienna).

[3] Pouwels S (2013). Risk of fracture in patients with Parkinson's disease. Osteoporos. Int..

[4] Karadsheh MS (2015). Mortality and revision surgery are increased in patients with Parkinson's disease and fractures of the femoral neck. Clin. Orthop. Relat. Res..

[5] Bliemel C (2015). Impact of Parkinson's disease on the acute care treatment and medium-term functional outcome in geriatric hip fracture patients. Arch. Orthop. Trauma Surg..

[6] Walker RW, Chaplin A, Hancock RL, Rutherford R, Gray WK (2013). Hip fractures in people with idiopathic Parkinson's disease: incidence and outcomes. Mov. Disord..

[7] Galbusera F (2018). Surgical treatment of spinal disorders in Parkinson's disease. Eur. Spine J..

[8] Oichi T (2017). Mortality and morbidity after spinal surgery in patients with Parkinson's disease: a retrospective matched-pair cohort study. Spine J..

[9] Coomber R, Alshameeri Z, Masia AF, Mela F, Parker MJ (2017). Hip fractures and Parkinson's disease: a case series. Injury.

[10] Price CC (2015). Orthopedic surgery and post-operative cognitive decline in idiopathic Parkinson's disease: considerations from a pilot study. J. Parkinsons Dis..

[11] Yuasa T, Maezawa K, Nozawa M, Kaneko K (2013). Surgical outcome for hip fractures in patients with and without Parkinson's disease. J. Orthop. Surg. (Hong Kong).

[12] Chou TA (2020). The outcome for surgical fixation of distal radial fractures in patients with idiopathic Parkinson's disease: a cohort study. J. Orthop. Surg. Res..

[13] Kryzak TJ, Sperling JW, Schleck CD, Cofield RH (2010). Hemiarthroplasty for proximal humerus fractures in patients with Parkinson's disease. Clin. Orthop. Relat. Res..

[14] Muhlenfeld N (2019). Fractures in Parkinson's disease: injury patterns, hospitalization, and therapeutic aspects. Eur. J. Trauma Emerg. Surg..

[15] Burrus MT, Werner BC, Cancienne JM, Gwathmey FW, Brockmeier SF (2015). Shoulder arthroplasty in patients with Parkinson's disease is associated with increased complications. J. Shoulder Elbow Surg..

[16] Guide, A. S. R. *Adult Trauma*. https://surgeryreference.aofoundation.org/orthopedic-trauma/adult-trauma. (2020, April).

[17] Leiblein M, Verboket R, Marzi I, Wagner N, Nau C (2019). Nonunions of the humerus—treatment concepts and results of the last five years. Chin. J. Traumatol..

[18] Prommersberger KJ, Fernandez DL (2004). Nonunion of distal radius fractures. Clin. Orthop. Relat. Res..

[19] Chen W, Tang K, Tao X, Yuan C, Zhou B (2018). Clavicular non-union treated with fixation using locking compression plate without bone graft. J. Orthop. Surg. Res..

[20] Wiegand L, Bernstein J, Ahn J (2012). Fractures in brief: olecranon fractures. Clin. Orthop. Relat. Res..

[21] Zura R, Mehta S, Della Rocca GJ, Steen RG (2016). Biological risk factors for nonunion of bone fracture. JBJS Rev..

[22] Raglione LM, Sorbi S, Nacmias B (2011). Osteoporosis and Parkinson's disease. Clin. Cases Miner Bone Metab..

[23] Meals C, Meals R (2013). Hand fractures: a review of current treatment strategies. J. Hand Surg. Am..

[24] Strub B (2010). Intramedullary splinting or conservative treatment for displaced fractures of the little finger metacarpal neck? A prospective study. J. Hand Surg. Eur..

[25] Dacombe PJ, Amirfeyz R, Davis T (2016). Patient-reported outcome measures for hand and wrist trauma: is there sufficient evidence of reliability, validity, and responsiveness?. Hand (NY).

